# A Food Consumption-Based Diet Quality Score and Its Correlation With Nutrient Intake Adequacy Among Japanese Children

**DOI:** 10.7759/cureus.19337

**Published:** 2021-11-07

**Authors:** Tatsuya Koyama

**Affiliations:** 1 Department of Nutrition, Aomori University of Health and Welfare, Aomori, JPN

**Keywords:** japanese people, cross-sectional study, dietary reference intake for japanese, japanese food guide spinning top, pediatric diet quality

## Abstract

Objective: Diet quality indices reflect overall dietary patterns better than single nutrients or food groups. Focusing on the needs of children, this study developed a measure of adherence to the Japanese Food Guide Spinning Top (JFGST), which was developed by the Ministry of Health, Labour and Welfare and Ministry of Agriculture, Forestry and Fisheries, Japan, and determined the correlation between index scores and nutrient intake.

Research Methods and Procedures: This cross-sectional study was conducted among 48 children between six and nine years of age from a coastal town in the Kinki region of Japan. Data were collected between August 2012 and March 2013, including the 12-day diet records (three days over four seasons) of each participant. For children, adherence to the JFGST entails the consumption of five core food groups, including grain, fish and meat, vegetables, milk, and fruits (total possible score of 50). Spearman’s correlation coefficients were calculated to identify any correlations between JFGST scores and nutrient intake.

Results: The mean participant JFGST score was 25 ± 10. Higher JFGST scores were correlated with higher intake of monounsaturated fatty acids, n-6 polyunsaturated fatty acids, dietary fiber, potassium, calcium, magnesium, phosphorus, zinc, copper, vitamin A, vitamin D, riboflavin, pantothenate, and vitamin C. JFGST scores were also positively correlated with nutrient adequacy for calcium, magnesium, iron, zinc, copper, vitamin A, thiamine, riboflavin, vitamin B_6_, vitamin B_12_, folate, and vitamin C.

Conclusion: The index employed in this study may adequately express diet quality among Japanese children.

## Introduction

Dietary guidelines are created by translating nutrition science into public health advice. Typically, they are designed to promote the populational adoption of dietary patterns that are known to minimize the risks of nutritional deficiency and chronic disease [1]. For children and adolescents, dietary guidelines encourage the intake of particular types of foods in appropriate amounts in order to support growth, meet the body’s nutritional needs, and minimize the potential for acute and chronic diet-related illnesses. When assessing populational nutrition, public health professionals require tools to help them create dietary guidelines in the form of a recommendations package [2]. Specifically, such tools will help these professionals monitor populational intakes against dietary guidelines, evaluate public health nutrition interventions, and validate dietary guidelines in terms of whether they promote eating patterns that are associated with positive health outcomes.

Over the last decade, various researchers have developed indices to measure the level of adherence to dietary guidelines, but most of these were designed for use with adult populations and in Western contexts [3,4]. Meanwhile, a small number of Japanese researchers have developed indices aimed at diet quality in adult populations. This includes an adherence score for Japanese dietary guidelines, which was validated against both nutrient intake and the risk of premature mortality [5,6]. However, this index must be adapted to adequately address the dietary issues of children, including alterations to the scoring criteria to reflect age-specific recommendations. In sum, there is currently a lack of diet indices that can accurately assess adherence to dietary guidelines in pediatric populations, with even fewer built for use in the Japanese context [7].

Based on the above issues, this study developed a diet quality index for use in pediatric populations in Japan, specifically, to measure adherence to the JFGST. We subsequently investigated the correlations between scores from this index and nutrient adequacy among a sample comprising 48 children who were between six and nine years of age.

## Materials and methods

This cross-sectional study was conducted between August 2012 and March 2013. As mentioned, the participants included 48 children who were between six and nine years of age at baseline (August 2012). All participants lived in a coastal town in the Kinki region of Japan; the only exclusion criterion was residence outside this town. We conducted a dietary survey during the study period, with four waves in both 2012 and 2013, respectively, including August 2012 (summer), November 2012 (autumn), January 2013 (winter), and March 2013 (spring). In each season, the survey was conducted on three nonconsecutive days, two being weekdays and one being on a weekend.

The survey was conducted in accordance with the tenets of the Declaration of Helsinki using a study protocol that was approved by the Ethics Committee of the Faculty of Human Life Science, Osaka City University (approval number 19-05). Informed consent was obtained from the guardians of all participating children. The same guardians were asked to record the food these participants consumed. Specifically, home-based dietary intake was recorded by the guardian who most often prepared meals for each respective participant. This included all food and beverages consumed outside the school environment. Each guardian was provided with a manual in which to record dietary intake, record sheets, and a measuring utensil set containing a cup, spoons, and a kitchen scale. Concerning this last item, they were instructed to weigh all ingredients (solid foods and liquids, including drinks) provided to their respective participants whenever possible. In cases where participants dined out and weighing was therefore difficult, the guardians were asked to record the name of the restaurant, dish consumed by the participant, and any foods that were left unconsumed. The main items documented via the record sheets included names of dishes, names of foods or ingredients used in dish preparation, approximate volume of the consumed food (measured with any of the provided utensils or by counting, as suitable), and the weights of each ingredient, food, and/or dish. The guardians were also asked to submit packages of any prepared foods or snacks along with the record sheets to facilitate our estimation of the ingredient amounts. For school lunches, dietary intake was estimated through direct observation and by examining the menus prepared by school dietitians. The staff recorded the amount of food consumed by each participant by subtracting the amount leftover from the amount estimated based on the data provided in these menus.

While wearing light clothing and no shoes, participants were measured for body height and weight to the nearest 0.1 cm and 0.1 kg, respectively, at baseline (August 2012). The prevalence of childhood obesity was evaluated using the percentage of excess weight, as calculated using the following formula: ({actual weight − standard weight/standard weight} × 100). Participants were respectively categorized as overweight and underweight in cases where the percentage of excess weight was ≥20% and ≤-20%. The standard weight was calculated using an age- and sex-specific formula based on height and coefficients [8].

Dietary intake was estimated using both the Standard Tables of Food Composition in Japan [9] and the classification of food groups used in the National Health and Nutrition Survey Japan [10]. Nutrient intake values were energy-adjusted using the density method (i.e., percentage of energy for energy-providing nutrients and amount per 1000 kcal of energy for other nutrients).

Following Oba et al. [11], we developed a diet quality score representing adherence to the JFGST using information on daily dietary intake. Briefly, the JFGST provides serving standards for five categories of dishes, including grain dishes, fish and meat dishes (fish, meat, egg, and soybean dishes), vegetable dishes, milk (milk and milk products), and fruits [12]. The recommended intake amounts are four to five servings/day for grain dishes, three to four servings/day for fish and meat dishes, five to six servings/day for vegetable dishes, two to three servings/day for milk, and two servings/day for fruits. One serving of a grain dish contains 40 g of carbohydrates from foods classified as grains. One serving of a fish and meat dish contains 6 g of protein from foods classified as fish, meat, egg, and/or soybean. One serving of a vegetable dish contains 70 g of foods classified as vegetables, mushrooms, and/or algae. One serving of milk contains 100 mg of calcium from foods classified as a milk and/or dairy product. Finally, one serving of fruits corresponds to 100 g of foods classified as fruit.

A total JFGST score may range from 0 (lowest quality diet) to 50 (highest quality diet). This is obtained by summing the individual scores from each dish category (i.e., grain, fish and meat, vegetables, milk, and fruit dishes). In this study, participants received 10 points for any dish category in which they consumed the recommended number of servings, as listed above. Their scores were proportionately adjusted in cases where they exceeded or did not meet these recommendations. If a participant consumed less than the recommended number of servings in a given category, then their score was calculated using the following formula: 10 × (the consumed amount of serving)/(the lower limit of the recommended amount). If they consumed more than the recommended number of servings for a given category, then their score was calculated using the following formula: 10 - 10 × ([the consumed amount of servings - the upper limit of the recommended amount]/the upper limit of the recommended amount). The score was converted to zero in cases where this calculation produced a negative value due to excess servings.

Nutrient adequacy was evaluated for 13 nutrients (i.e., calcium(Ca), magnesium (Mg), iron (Fe), zinc(Zn), copper (Cu), vitamin A, thiamine, riboflavin, niacin, vitamin B6, vitamin B12, folate, and vitamin C) using a probabilistic approach [13]. Here, estimated average requirements (EARs) were set for each nutrient. In this approach, the probability of nutrient intake adequacy was calculated from a z-score as follows: z-score = (nutrient intake - EAR)/standard deviation of the EAR.

Spearman’s correlation coefficients were calculated to identify any correlations between JFGST scores and nutrient intake. All analyses were performed using IBM SPSS version 26 ( released 2019. Armonk, NY, USA: IBM Corp.). p-values < 0.05 (two-tailed) were considered indicative of statistical significance.

## Results

For boys, the mean (standard deviation) age, height, weight, and percentage of excess weight were 7.7 (1.2) years, 125.8 (9.1) cm, 25.4 (5.4) kg, and -2.7% (7.3) at baseline, respectively. For girls, the corresponding values were 7.9 (0.9) years, 123.9 (7.2) cm, 24.4 (5.5) kg, and -1.2% (13.6), respectively. There was only one overweight participant and one underweight participant in the entire cohort.

The mean energy intake was 1707 (334) kcal/day. The mean JFGST score was 25 (10). The total JFGST score was positively correlated with the intake of monounsaturated fatty acids (MUFA), n-6 polyunsaturated fatty acids (PUFA), dietary fiber, potassium (K), Ca, Mg, phosphorus (P), Zn, Cu, vitamin A, vitamin D, riboflavin, pantothenate, and vitamin C (Table 1). The score for the grain dishes was positively correlated with the intake of carbohydrates and Cu but negatively correlated with the intake of total fat, saturated fatty acids (SFA), MUFA, PUFA, n-6 PUFA, and niacin. The fish and meat dishes score was positively correlated with the intake of proteins and Zn but negatively correlated with the intake of dietary fiber, vitamin E, and thiamine. The vegetable dishes score was positively correlated with the intake of dietary fiber, K, Ca, Mg, P, iron, zinc, Cu, vitamin A, vitamin D, vitamin K, thiamine, vitamin B6, folate, pantothenate, and vitamin C. The milk score was positively correlated with the intake of proteins, SFA, Ca, P, Zn, vitamin A, riboflavin, and pantothenate, but negatively correlated with the intake of niacin. Finally, the fruits score was positively correlated with the intake of n-3 PUFA, dietary fiber, K, Mg, vitamin D, vitamin E, vitamin K, thiamine, vitamin B6, folate, and vitamin C.

**Table 1:** Correlation between Japanese Food Guide Spinning Top (JFGST) scores (dependent variable) and nutrient intake (independent variables) (n=48) SFA: saturated fatty acids; MUFA: monounsaturated fatty acids; PUFA: polyunsaturated fatty acids; Na: sodium; K: potassium; Ca: calcium; Mg: magnesium; P: phosphorus; Fe: iron; Zn: zinc; Cu: copper

	Mean ± standard deviation			Spearman’s correlation coefficients											
				JFGST score (0-50)		Grain dishes score (0-10)		Fish and meat dishes score (0-10)		Vegetable dishes score (0-10)		Milk score (0-10)		Fruits score (0-10)	
JFGST score (0-50)	29.8	±	3.5	1.00	-	0.42	***	0.07		0.27		0.19		-0.12	
Grain dishes score (0-10)	8.0	±	1.0	0.19		0.07		1.00	-	0.03		0.18		-0.08	
Fish and meat dishes score (0-10)	8.3	±	0.6	0.72	^***^	0.27		0.03		1.00	-	0.27		0.32	^*^
Vegetable dishes score (0-10)	4.8	±	1.4	0.60	^***^	0.19		0.18		0.27		1.00	-	-0.15	
Milk score (0-10)	6.2	±	1.6	0.46	^**^	-0.12		-0.08		0.32	^*^	-0.15		1.00	-
Fruits score (0-10)	2.5	±	1.8	0.51	^***^	0.68	^***^	0.12		0.38	^**^	0.25		0.09	
Protein (%E)	13.6	±	1.2	0.19		-0.27		0.37	^**^	0.10		0.29	^*^	0.09	
Total fat (%E)	28.0	±	3.4	-0.20		-0.45	^**^	-0.01		-0.18		0.14		-0.03	
SFA (%E)	8.8	±	1.4	-0.15		-0.31	^*^	-0.03		-0.22		0.31	^*^	-0.17	
MUFA (%E)	9.7	±	1.4	-0.34	^*^	-0.53	^***^	-0.05		-0.28		-0.03		0.00	
PUFA (%E)	5.2	±	0.7	-0.27		-0.34	^*^	0.00		-0.16		-0.24		0.22	
n-3 PUFA (%E)	0.8	±	0.2	-0.01		-0.28		0.15		-0.04		-0.22		0.44	^**^
n-6 PUFA (%E)	4.3	±	0.6	-0.33	^*^	-0.33	^*^	-0.02		-0.17		-0.23		0.12	
Carbohydrate (%E)	58.4	±	3.7	0.12		0.48	^**^	-0.06		0.13		-0.21		0.00	
Dietary fiber (g/1000kcal)	5.3	±	1.0	0.34	^*^	-0.05		-0.30	^*^	0.60	^***^	-0.07		0.43	^**^
Na (mg/1000kcal)	1726	±	251	-0.03		-0.24		-0.20		0.11		-0.06		0.02	
K (mg/1000kcal)	986	±	129	0.49	^***^	-0.18		-0.05		0.52	^***^	0.13		0.59	^***^
Ca (mg/1000kcal)	297	±	62	0.47	^**^	0.06		-0.11		0.31	^*^	0.60	^***^	0.08	
Mg (mg/1000kcal)	101	±	13	0.42	^**^	0.00		-0.13		0.54	^***^	0.13		0.41	^**^
P (mg/1000kcal)	498	±	52	0.41	^**^	-0.07		0.24		0.33	^*^	0.47	^**^	0.05	
Fe (mg/1000kcal)	3.0	±	0.5	0.10		-0.22		-0.09		0.31	^*^	-0.08		0.31	
Zn (mg/1000kcal)	4.1	±	0.3	0.36	^*^	0.03		0.44	^**^	0.29	^*^	0.46	^**^	-0.09	
Cu (mg/1000kcal)	0.5	±	0.1	0.36	^*^	0.33	^*^	-0.06		0.50	^***^	-0.03		0.16	
Vitamin A (μg/1000kcal)	233	±	69	0.38	^**^	-0.16		-0.22		0.46	^**^	0.34	^*^	0.19	
Vitamin D (μg/1000kcal)	2.5	±	1.1	0.33	^*^	-0.10		0.18		0.30	^*^	-0.01		0.45	^**^
Vitamin E (mg/1000kcal)	3.1	±	0.6	0.03		-0.16		-0.31	^*^	0.19		-0.16		0.40	^**^
Vitamin K (μg/1000kcal)	70	±	25	0.24		-0.06		-0.09		0.38	^**^	0.05		0.31	^*^
Thiamine (mg/1000kcal)	0.45	±	0.07	0.28		-0.08		-0.34	^*^	0.36	^*^	0.09		0.36	^*^
Riboflavin (mg/1000kcal)	0.64	±	0.11	0.37	^*^	0.05		-0.03		0.22		0.50	^***^	0.00	
Niacin (mg/1000kcal)	6.2	±	1.5	-0.24		-0.35	^*^	0.09		-0.07		-0.30	^*^	0.09	
Vitamin B_6_ (mg/1000kcal)	0.46	±	0.08	0.26		-0.22		0.12		0.41	^**^	-0.07		0.40	^**^
Vitamin B_12_ (μg/1000kcal)	2.4	±	0.8	0.18		0.01		0.26		0.12		0.05		0.09	
Folate (μg/1000kcal)	103	±	26	0.19		-0.10		-0.11		0.47	^**^	-0.17		0.38	^**^
Pantothenate (mg/1000kcal)	2.7	±	0.3	0.45	^**^	-0.06		0.19		0.29	^*^	0.52	^***^	0.13	
Vitamin C (mg/1000kcal)	29	±	11	0.45	^**^	-0.09		0.01		0.52	^***^	-0.09		0.70	^***^

The total JFGST score was positively correlated with nutrient intake adequacy for Ca, Mg, Fe, Zn, Cu, vitamin A, thiamine, riboflavin, vitamin B6, vitamin B12, folate, and vitamin C (Table 2). The grain dishes score was positively correlated with nutrient intake adequacy for Mg, Cu, thiamine, and folate. The vegetable dishes score was positively correlated with nutrient adequacy for Ca, Mg, Fe, Zn, Cu, vitamin A, thiamine, vitamin B6, folate, and vitamin C. The milk score was positively correlated with nutrient adequacy for Ca, Mg, Fe, Zn, vitamin A, thiamine, riboflavin, and vitamin B12. Lastly, the fruits score was positively correlated with nutrient adequacy for Mg, Fe, thiamine, vitamin B6, folate, and vitamin C.

**Table 2:** Correlation between Japanese Food Guide Spinning Top (JFGST) scores and nutrient adequacy (%) (n=48) Ca: calcium; Mg: magnesium; Fe: iron; Zn: zinc; Cu: copper

	Mean ± standard deviation			Spearman’s correlation coefficients													
				JFGST score (0-50)		Grain dishes score (0-10)		Fish and meat dishes score (0-10)		Vegetable dishes score (0-10)			Milk score (0-10)		Fruits score (0-10)		
Ca	49.1	±	46.6	0.56	***	0.28		0.12			0.29	*	0.60	***	0.02		
Mg	87.0	±	27.8	0.70	***	0.31	*	0.11			0.61	***	0.38	*	0.34	*	
Fe	40.8	±	38.7	0.54	***	0.14		0.15			0.47	**	0.35	*	0.30	*	
Zn	99.9	±	0.4	0.56	***	0.26		0.27			0.32	*	0.44	**	0.14		
Cu	96.4	±	8.6	0.42	**	0.59	***	0.12			0.38	*	0.03		0.11		
Vitamin A	66.9	±	33.0	0.59	***	0.09		-0.04			0.55	***	0.52	***	0.15		
Thiamine	48.5	±	39.1	0.58	***	0.36	*	-0.12			0.48	**	0.36	*	0.30	*	
Riboflavin	73.1	±	31.7	0.39	**	0.05		0.01			0.21		0.51	***	0.03		
Niacin	88.0	±	20.3	0.16		-0.03		0.21			0.20		-0.07		0.25		
Vitamin B_6_	58.4	±	38.7	0.65	***	0.12		0.23			0.60	***	0.25		0.46	*	
Vitamin B_12_	100.0	±	0.0	0.32	*	-0.02		0.21			0.26		0.38	**	0.05		
Folate	91.5	±	18.4	0.68	***	0.31	*	0.01			0.79	***	0.27		0.38	*	
Vitamin C	29.1	±	41.2	0.68	***	0.09		0.07			0.65	***	0.15		0.72	***	

## Discussion

This study examined the relationship between food-based diet quality scores and nutrient intake adequacy among Japanese children who were between six and nine years of age. The results showed that JFGST-based diet scores were positively correlated with favorable dietary intake patterns, including nutrient intake adequacy for Ca, Mg, Fe, Zn, Cu, vitamin A, thiamine, riboflavin, vitamin B6, vitamin B12, folate, and vitamin C. These findings suggest that the JFGST-based score may adequately reflect overall diet quality in Japanese children.

There is no literature on the validity and reliability of JFGST scores in Japanese children. We examined the correlation between JFGST score and nutrient intake in each season and similar results were observed in each season. Therefore, we confirmed the validity and reliability of JFGST score in Japanese children. 

Dietary guidelines and relevant indices are important factors for objectively ensuring and measuring overall health. For example, a previous prospective cohort study showed that better adherence to dietary guidelines was associated with a lower risk of premature mortality among women [3]. Based on data from the 2012 National Health and Nutrition Survey Japan, another study found that better guideline adherence was inversely associated with body mass index, waist circumference, systolic blood pressure, and high-density lipoprotein cholesterol levels [9]. In the current study, participants generally returned low scores for both vegetable and fruit intake and showed low nutrient intake adequacy for vitamin C. Indeed, the vegetable and fruit dishes scores were positively correlated with vitamin C intake. This suggests that increased vegetable and fruit intake may be important for improving overall diet quality in the target population.

We found no correlation between the JFGST score and energy-providing nutrient balance. For children between six and nine years of age, the Dietary Reference Intakes for Japanese 2020 guidelines recommend respective energy intake percentages of 13-20%, 20-30%, ≤10%, and 50-65% for proteins, total fats, saturated fatty acids, and carbohydrates, respectively [8]. Individuals in Japan tend to consume less fats and more carbohydrates than individuals in Western countries [10]. Meanwhile, a previous study showed that children tend to derive higher proportions of their energy from fats and lower proportions of their energy from carbohydrates when compared to adults [7]. However, there is currently a lack of evidence concerning the association between macronutrient intake and intake of other nutrients among Japanese children, thus highlighting the need for continued research to clarify how macronutrient intake influences micronutrient adequacy in this population.

This study also had some limitations. First, it did not recruit a nationally representative sample, as all participants resided in the same town in the Kinki region. This may have introduced selection bias, and also limits generalizability to other populations. Second, the sample size was relatively small. Third, sex, height, and weight were the only participant characteristics considered in this study. As such, this study could not test for associations between the JFGST score and socioeconomic status.

## Conclusions

This cross-sectional study examined dietary intake among Japanese children between six and nine years of age using an index designed to reflect JFGST recommendations. We found a correlation between their overall JFGST-based diet quality scores and age-adjusted nutrient intake adequacy. In sum, these findings indicate that the JFGST is suitable for assessing dietary quality among Japanese children. Guardians and school staff may use this information to ensure that children practice well-balanced dietary habits in both the home and school environments.
